# Recovery of metagenome-assembled microbial genomes from a full-scale biogas plant of food waste by pacific biosciences high-fidelity sequencing

**DOI:** 10.3389/fmicb.2022.1095497

**Published:** 2023-01-09

**Authors:** Fan Jiang, Qiang Li, Sen Wang, Ting Shen, Hengchao Wang, Anqi Wang, Dong Xu, Lihua Yuan, Lihong Lei, Rong Chen, Boyuan Yang, Yu Deng, Wei Fan

**Affiliations:** ^1^Guangdong Laboratory for Lingnan Modern Agriculture (Shenzhen Branch), Genome Analysis Laboratory of the Ministry of Agriculture and Rural Affairs, Agricultural Genomics Institute at Shenzhen, Chinese Academy of Agricultural Sciences, Shenzhen, Guangdong, China; ^2^Biogas Institute of Ministry of Agriculture and Rural Affairs, Chengdu, Sichuan, China; ^3^Key Laboratory of Development and Application of Rural Renewable Energy, Ministry of Agriculture and Rural Affairs, Chengdu, Sichuan, China

**Keywords:** food waste, metagenome, PacBio HiFi reads, full-scale biogas plants, full-length 16S rRNA genes

## Abstract

**Background:**

Anaerobic digestion (AD) is important in treating of food waste, and thousands of metagenome-assembled genomes (MAGs) have been constructed for the microbiome in AD. However, due to the limitations of the short-read sequencing and assembly technologies, most of these MAGs are grouped from hundreds of short contigs by binning algorithms, and the errors are easily introduced.

**Results:**

In this study, we constructed a total of 60 non-redundant microbial genomes from 64.5 Gb of PacBio high-fidelity (HiFi) long reads, generated from the digestate samples of a full-scale biogas plant fed with food waste. Of the 60 microbial genomes, all genomes have at least one copy of rRNA operons (16S, 23S, and 5S rRNA), 54 have ≥18 types of standard tRNA genes, and 39 are circular complete genomes. In comparison with the published short-read derived MAGs for AD, we found 23 genomes with average nucleotide identity less than 95% to any known MAGs. Besides, our HiFi-derived genomes have much higher average contig N50 size, slightly higher average genome size and lower contamination. GTDB-Tk classification of these genomes revealed two genomes belonging to novel genus and four genomes belonging to novel species, since their 16S rRNA genes have identities lower than 95 and 97% to any known 16S rRNA genes, respectively. Microbial community analysis based on the these assembled genomes reveals the most predominant phylum was *Thermotogae* (70.5%), followed by *Euryarchaeota* (6.1%), and *Bacteroidetes* (4.7%), and the most predominant bacterial and archaeal genera were *Defluviitoga* (69.1%) and *Methanothrix* (5.4%), respectively. Analysis of the full-length 16S rRNA genes identified from the HiFi reads gave similar microbial compositions to that derived from the 60 assembled genomes.

**Conclusion:**

High-fidelity sequencing not only generated microbial genomes with obviously improved quality but also recovered a substantial portion of novel genomes missed in previous short-read based studies, and the novel genomes will deepen our understanding of the microbial composition in AD of food waste.

## Introduction

In recent years, rapid growth of population and economic activities are producing more and more municipal solid wastes (Li et al., 2017). Food waste has caused increasing public concerns, with the global production estimated to be around 1.4 billion tons per year, accounting for 30–50% of total municipal solid wastes (Kiran et al., 2014; Wong et al., 2018; Wu et al., 2021). When improperly disposed, food waste releases a lot of foul-smelling odor and leachate due to its high content of volatile solids (85–95%) and moisture (66–80%; Kim et al., 2016; Fisgativa et al., 2017; Zan et al., 2018). On the other hand, food waste is usually rich in carbohydrates, proteins, lipids, and minerals, which makes it a potential valuable source for production of renewable energy (Paritosh et al., 2017; Antonopoulou et al., 2019). At the same time, anaerobic digestion (AD) is considered as one of the most cost-effective and eco-friendly technologies for both renewable energy production and waste disposal, and to date has been widely used for treating of food waste (Xu et al., 2018; Garcia-Depraect et al., 2022; Zhang S. et al., 2022).

Anaerobic digestion is performed by a highly complex consortium of bacteria and archaea, and the process includes four sequential metabolic steps of hydrolysis, acidogenesis, acetogenesis, and methanogenesis (Angenent et al., 2004; Hassa et al., 2018). The first three steps are predominantly fulfilled synergistically by fermentative bacteria, while the last step is carried out by methanogenic archaea (Schnürer, 2016). To investigate the microbial communities in AD, 16S rRNA gene amplicon analysis has been widely used with the development of high-throughput short-read sequencing technologies (Li et al., 2022; Zhang S. et al., 2022). In recent years, the genome-centric metagenome analysis has been used for investigating the complex microbial communities in AD, and recovered a large number of metagenome-assembled genomes (MAGs; Treu et al., 2016; Campanaro et al., 2018, 2020). In 2020, Campanaro et al. constructed 1,401 MAGs from 134 publicly available metagenomes derived from different biogas plants, and then in 2021, Ma et al. constructed 2,426 MAGs from 56 full-scale biogas plants with different feedstocks (Campanaro et al., 2020; Ma et al., 2021), and these efforts greatly deepen the understanding of microbial communities of AD and provide a huge number of microbial genomes for uncultured microbes in AD.

Genome-centric metagenome analysis have facilitated the recovery of microbial genomes for novel organisms residing in AD microbiomes, such as the members of the archaeal phylum *Bathyarchaeota*, which were proposed to contribute to hydrolysis and subsequent fermentation of organic materials within the process of biogas production (Evans et al., 2015; Maus et al., 2018). However, due to the technical limitation of short-read sequencing, these assembled genomes often fragmented and with some wrong contigs introduced by binning algorithms, and it will partly limit their applications. Recently, the use of highly accurate HiFi long reads and the related assemblers have greatly improved the assembly quality of MAGs, which even generated hundreds of circular genomes (Bickhart et al., 2022; Feng et al., 2022). Besides, high proportion of novel genomes were generated, which recovered 89 novel genomes (26% of the total assembled genomes) from the chicken gut microbiomes by using 332 Gb of HiFi reads (Zhang Y. et al., 2022). In addition, HiFi long reads also enabled the analysis of full-length 16S rRNA genes covering all hypervariable regions, which could provide more accurate taxonomic classification for microorganisms (Liang et al., 2022). In this study, we used the HiFi long reads to construct the microbial genomes for microorganisms in AD of food waste, and analyzed the full-length 16S rRNA genes identified from the sequenced long reads to investigate the microbial communities.

## Materials and methods

### Full-scale anaerobic digester of food waste

A full-scale up-flow solid anaerobic digester was selected from a food waste treatment plant in Chengdu, China, which can treat 100 tons of food waste every day. Before entering the digesters, the food waste will undergo a complex process of pretreatment: firstly, after sorting out the impurities such as glass bottles and bowls, the food waste was broken in the pulping machine, and the plastic and woven bags were screened out by the residue liquid separator; secondly, the treated food waste was transported to the sterilization tank for heating, heat preservation (80–95°C) and sterilization; finally, three-phase separation was performed, and the residue phase was used to make organic fertilizer, the oil phase was used to extract industrial diesel oil, and only the liquid phase was pumped into the anaerobic digesters to produce biogas. Because the temperature of the liquid phase is high (60–70°C) after the three-phase separation, it should be cooled to 40–45°C in the buffer tank before entering the digester. At last, the digestate will be discharged after it meets the discharge standard through further water treatment process, and the produced biogas will be used as fuel for pretreating the food waste.

The effective volume of the sampling digester is 3,500 m^3^, and the hydraulic retention time of the liquid phase is 37–41 days, the organic loading rate is 2.48 kg chemical oxygen demand (COD) m^−3^ d^−1^. The fermentation temperature is 37°C, the pH of the digestate is 7.3, and the daily gas production is on average 6,000 m^3^. At the day of sampling, the methane, carbon dioxide, and nitrogen contents were 61.1, 36.6, and 2.3% (v/v), respectively. The average concentration of acetate, propionate, and butyrate were 38.7, 18.8, and 20.0 mg L^−1^, respectively. The average concentration of total ammonium nitrogen was 1,224.7 mg L^−1^. The other physicochemical characteristics of the fermented digestate and the fed liquid phase from the food waste are shown in Supplementary Table S1.

### Sample collection

Digestate samples were collected from the sampling valve of the anaerobic digester. Before sampling, the reactor content was stirred and the sampling valve was opened for 5 min to flush the sampling valve and tubes. Approximately, 3,000 ml of digestate was collected into six sterile, gastight tubes (500 ml) and transported to the laboratory immediately. Upon arrival in the laboratory, 1,000 ml subsamples were homogenized and centrifuged at 9,000 rpm for 30 min (at 4°C), and after washing with phosphate buffered saline (pH 7.2) and centrifuging again under the same conditions, the pellet was collected in 50 ml sterile tubes and stored at −80°C before DNA extraction. Remaining 2,000 ml subsamples were stored at −20°C before physicochemical analysis. Biogas samples were collected with gas sample bags and stored at room temperature.

### Physicochemical analysis

The total solid (TS), total ammonia nitrogen (TAN), chemical oxygen demand (COD), C content, and N content were measured according to APHA Standard Protocols (Simmons et al., 1998). Volatile fatty acids (VFAs) were analyzed by a gas chromatography (GC7890, Agilent, United States) with a flame-ionization detector and a capillary column (DB-FFAP 0.25 × 0.5 × 30), the initial temperature of column was 80°C, raise it to 180°C at a speed of 28°C s^−1^, and keeping 1 min, and then, raise it to 220°C at a speed of 40°C s^−1^ and keeping 1 min, the temperature of injection port and detector were 210 and 230°C, respectively. Injection volume was 1 μl and split ratio was 1:10. Nitrogen was the carrier gas and the flow rate was 35 cm s^−1^, the flow rate of hydrogen, air and tail blowing were 30, 300, and 38 ml min^−1^, respectively. VFAs were carried out with external standard method. The biogas composition was analyzed by a gas chromatography (GC7820A, Agilent, United States) with a thermal-conductivity detector and stainless steel column (1/8 × 3 m) packed with supporter (Porapak Q), hydrogen as the carrier gas and the flow rate was 27 ml min^−1^, and temperatures for the column, injection port, and detector were 65, 120, and 130°C, respectively. Gas content was quantified with the area normalization method. pH values were measured with pH meters (AS ONE AS600, Japan).

### DNA extraction, library preparation, and sequencing

Genomic DNA from the sample was extracted using DNeasy PowerSoil Pro kit (ref. 47014; QIAGEN, Germany) according to the manufacturer’s instructions. The integrity of extracted DNA was checked on 0.8% (w/v) agarose gel with GelRed nucleic acid gel stain (cat. no. 41003; Biotium, United States). The purity and quantity of the extracted DNA was assessed using Nanodrop 2000 (Thermo Fisher Scientific, United States) and Qubit 4.0 (Thermo Fisher Scientific, United States). Then, the high integrity (with obvious concentrated electrophoresis band >15 Kb in size) and quality (A260/280 1.8–2.0, dsDNA concentration > 100 ng μl^−1^) DNA samples were used for library construction. For Pacific Biosciences (PacBio) Hifi sequencing, the DNA samples were fragmented into 15–20 Kb insets using g-TUBEs (Covaris, United States), and the sequencing library was constructed using SMRTbell Express Template Prep Kit 2.0 (PacBio, United States). Then, three SMRT cells of high-fidelity long reads were generated on PacBio Sequel II system with Circular Consensus Sequence (CCS) mode (PacBio, United States).

### Metagenome assembly and binning

**Figure 1:**
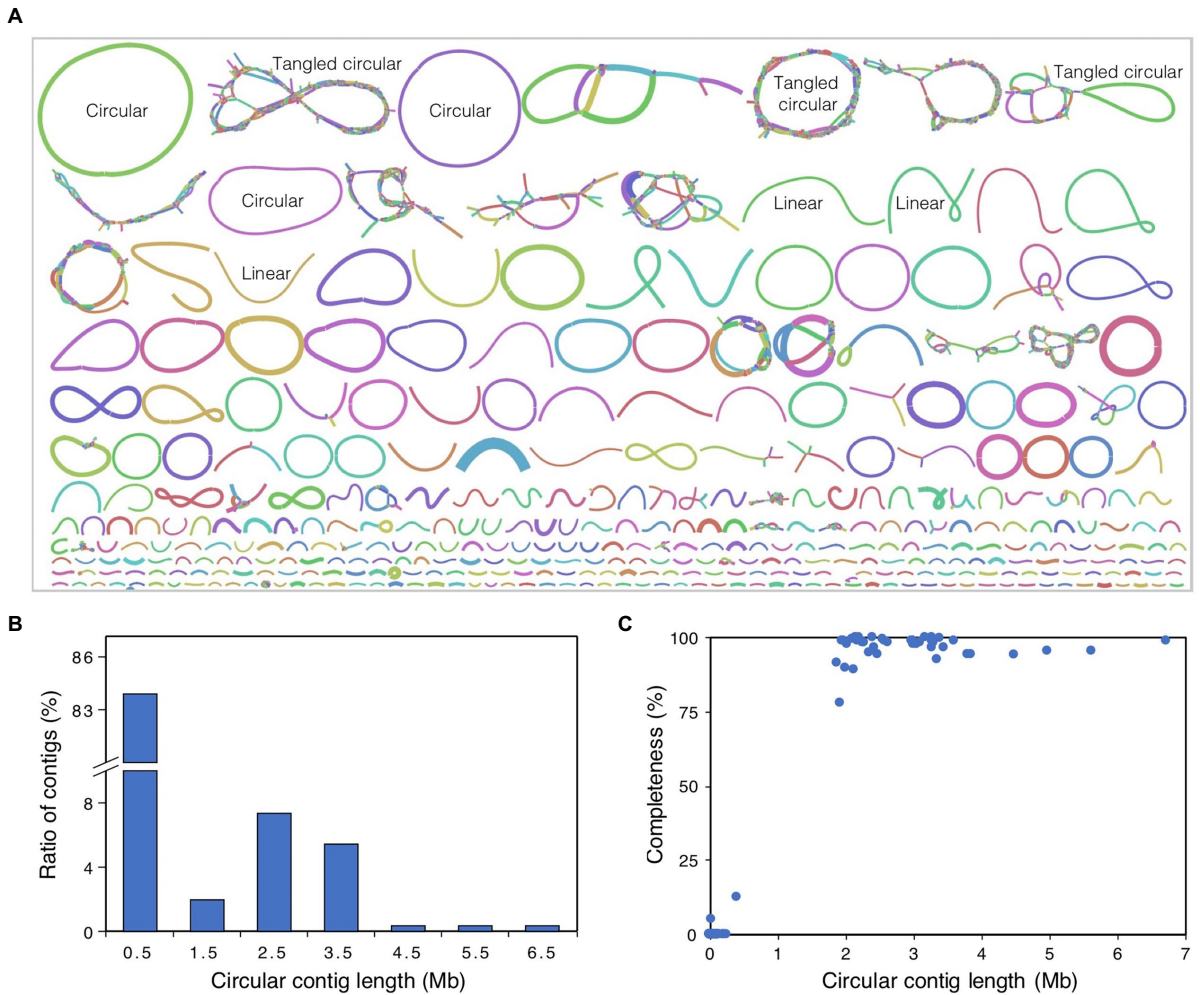
Overview of metagenome assembly. **(A)** Graphic display of the assembled contigs. The graph is drawn by Bandage, and the colors for different contigs are randomly assigned by the software. The line length is in proportion to contig length, and the line width is in proportion to contig coverage depth. Based on the structure of different contigs or contig groups, the contigs were divided into three types, circular contigs, tangled circular contigs, and linear contigs, and some examples were labeled in the figure. Circular contig, one single contig with head and tail concatenated; tangled circular contigs, many linear contigs link to each other and form a big circular or other complex structure; linear contig, one single contig with no link to other contigs. **(B)** Length distribution of circular contigs. **(C)** Relationship plot of completeness with contig length for all circular contigs. Completeness value of circular contigs was estimated using CheckM.

The raw HiFi sequencing reads were filtered by requiring a read length over 1 Kb and an average read accuracy over 99%, which resulted in a total of 64.6 Gb of high-quality reads from all three SMRT cells. To improve the assembly for less abundant species, all reads were pooled together for co-assembly. Then, Hifiasm-meta r058 (Feng et al., 2022) was used to assemble all HiFi reads into contigs with parameter “--force-preovec.” By viewing the contig linkages from the resulted GFA files with Bandage v0.8.1 (Wick et al., 2015), we observed that most of the assembled contigs can be divided into three types (Figure 1A): (1) circular contig, one single contig with head and tail concatenated; (2) tangled “circular” contigs, many linear contigs linked into a big circular or other complex structure; and (3) linear contig, one single contig with no link to other contigs. Then, the circular contigs were left alone, and each tangled “circle” was re-assembled by Hifiasm-meta r058 (Feng et al., 2022) with default parameters, using the fragmental contigs that constructed the “circle” as input reads, and resulted seven extra circular contigs. Finally, we obtained a total of 292 circular contigs, including 47 circular contigs with contig length longer than 1 Mb. In addition, plasmid and viral genomes were identified using viralVerify v1.1^1^ with parameter “--hmm nbc_hmms.hmm” from these circular contigs.

To group the separate linear contigs into metagenome-assembled genomes (MAGs), a binning algorithm MetaBAT2 v2.12.1 (Kang et al., 2019) was used, with removing all circular and tangled “circular” contigs from the assembled results. MetaBAT2 was used with parameter “-minContig 2000,” which resulted in a total of 28 non-circular MAGs.

The completeness (Cp) and contamination (Ct) of all circular contigs and non-circular MAGs were estimated using the “Lineage_wf” workflow of CheckM v1.07 (Parks et al., 2015) with options “lineage_wf-t 20-x fa.” According to these results, the microbial genome assemblies with three quality ranks were adopted: near complete (Cp ≥ 90% and Ct < 5%), high quality (Cp ≥ 70% and Ct < 10%), and medium quality (Cp ≥ 50% and Ct < 10%), including a total of 47 circular contigs and 28 non-circular MAGs.

### Construction of non-redundant microbial genome assemblies

De-replication of all filtered microbial genome assemblies (near-complete, high-quality, and medium-quality) were performed using Mash v2.2 (Ondov et al., 2016) on the entire genome sequences with very permissive parameters “dist-d 0.05” (Campanaro et al., 2020), which could cluster these genomes into different groups. To determine the representative genome for each group, a more precise analysis was performed applying the genome-wide average nucleotide identity (ANI; Varghese et al., 2015). When two genomes showed ANI value ≥95% and genome coverage ≥50% for both of them, they were considered to belong to the same species, and the genome with higher CheckM score (Cp—5 × Ct) was selected and retained. After many runs of comparison and selection, the representative genomes for each group were obtained, and other genomes were taken as redundant and removed. Finally, a total of 60 non-redundant microbial genomes were generated, comprising 39 circular and 21 non-circular genomes.

We used INFERNAL v1.1.2 (Nawrocki and Eddy, 2013) with parameters “cmscan --rfam --cut_ga --nohmmonly --cpu 10” to identify rRNA genes, tRNAscan-SE v2.0.3 (Chan et al., 2021) with parameters “-G-H” to identify tRNA genes. Taxonomic annotation of these genomes was performed using the GTDB-Tk v2.1.0 (Chaumeil et al., 2019, 2022) based on the GTDB database version r207 (Parks et al., 2022), with the parameter of “classify_wf-x fa.” In addition, 16S rRNA genes from each genome were also used for taxonomic annotation, with Ribosomal Database Project (RDP) Classifier v2.11 (Wang et al., 2007) to classify the genomes at lower taxonomic ranks, and BLAST alignments to the Silva database version r138 (Quast et al., 2013) were further used to validate the novelty of these taxonomic units. The phylogenetic tree of these genomes was constructed using FastTree v2.1.11 (Price et al., 2010) based on the 16S rRNA genes that retrieved from these genomes. To calculate the sequencing depth for the non-redundant genomes, high-quality HiFi reads were aligned back to these genomes using Minimap2 v2.20 (Li, 2018) with parameters “-x map-hifi-a -t 60,” and the resulted SAM format files were converted to sorted BAM format files by Samtools v1.3 (Li et al., 2009). Then, the depth was calculated using the script jgi_summarize_bam_contig_depths in MetaBAT2 according a previous study (Feng et al., 2022).

### Diversity analysis

To determine whether the real microbial composition of AD can be recovered from the assembled genomes, a 16S rRNA gene based microbial community analysis was performed. We identified the full-length 16S rRNA genes from high-quality HiFi reads using INFERNAL v1.1.2 (Nawrocki and Eddy, 2013) with parameters “cmscan --rfam --cut_ga --nohmmonly --cpu 48,” and the identified 16S rRNA genes with length < 1,200 or > 1,700 bp were filtered out. The average base error rate of each 16S rRNA gene was calculated, and the sequences with average accuracy less than 99% (Q20) were removed. As a result, a total of 54,135 16S rRNA genes were identified. Then, the RDP Classifier v2.11 (Wang et al., 2007) was used to classify the 16S rRNA genes, and the relative abundance of each taxonomic units was calculated by adjusting the gene copy number for 16S rRNA gene sequences according the database rrnDB v5.8 (Stoddard et al., 2015).

## Results and discussion

### Assembly of 47 complete circular microbial genomes and 118 viral genomes from high-fidelity long reads

To obtain a high-quality metagenome assembly for AD of food waste, a total of 64.6 Gb of PacBio HiFi reads were generated from three SMRT cells, with the read N50 size of 14.3 Kb and N90 size of 7.3 Kb (Table 1), and the average base accuracy of 99.94%. Then, we assembled the HiFi reads into contigs with Hifiasm-meta (Feng et al., 2022) and generated a total contig length of 924.9 Mb, with the N50 size of 87.7 Kb and N90 size of 22.2 Kb (Table 1), which were much longer than those assembled from Illumina short reads (Ma et al., 2021). By viewing the contig linkages from the resulted GFA file with Bandage (Wick et al., 2015), the contigs can be classified into three types of circular contig, tangled “circular” contigs and linear contig (Figure 1A), and a tangled structure contains many different strains of one species, while a circular or linear structure represents only one single microbial strain of one species.

**Table 1:** Statistics of sequencing reads and assembled contigs.

Statistics	Sequencing reads	Assembled contigs
Total number	5,833,109	17,715
Total length (bp)	64,564,585,452	924,882,211
Maximum (bp)	67,387	6,716,598
Minimum (bp)	1,000	1,390
N50 (bp)	14,254	87,732
N60 (bp)	13,140	59,332
N70 (bp)	12,074	43,546
N80 (bp)	10,854	32,503
N90 (bp)	7,310	22,219

The assembled metagenome contains 292 circular contigs with a total length of 146.0 Mb, which accounts for 15.8% of the total assembly length. The length distribution analysis of these circular contigs revealed that 245 contigs (83.9% of total circular contigs) were shorter than 1.0 Mb (Figure 1B), and 228 contigs were even shorter than 100 Kb. The other 47 circular contigs ranged from 1.0 to 6.7 Mb, and 37 contigs with lengths of 2.0 to 4.0 Mb accounted for 69.9% of the total length of circular contigs (Figure 1B). To determine whether the circular contigs are complete genomes, CheckM analysis was performed using the collocated sets of ubiquitous and single-copy genes within a phylogenetic lineage (Parks et al., 2015). The results revealed that the circular contigs longer than 1.0 Mb showed an average completeness of 97%, and 38, 6, and 3 of them have completeness >95%, 90–95%, and < 90%, respectively (Figure 1C). However, the completeness of the circular contigs shorter than 1.0 Mb was <15%, and 243 of them have completeness of 0% (Figure 1C). Then, viralVerify was used to classify these short circular contigs, and 31, 118, and 92 of them were classified as plasmid, virus, and uncertain, respectively, indicating that most of the short circular contigs were not genomic fragments derived from microbial genomes.

### Capacity of the assemblage of HiFi genomes

**Figure 2:**
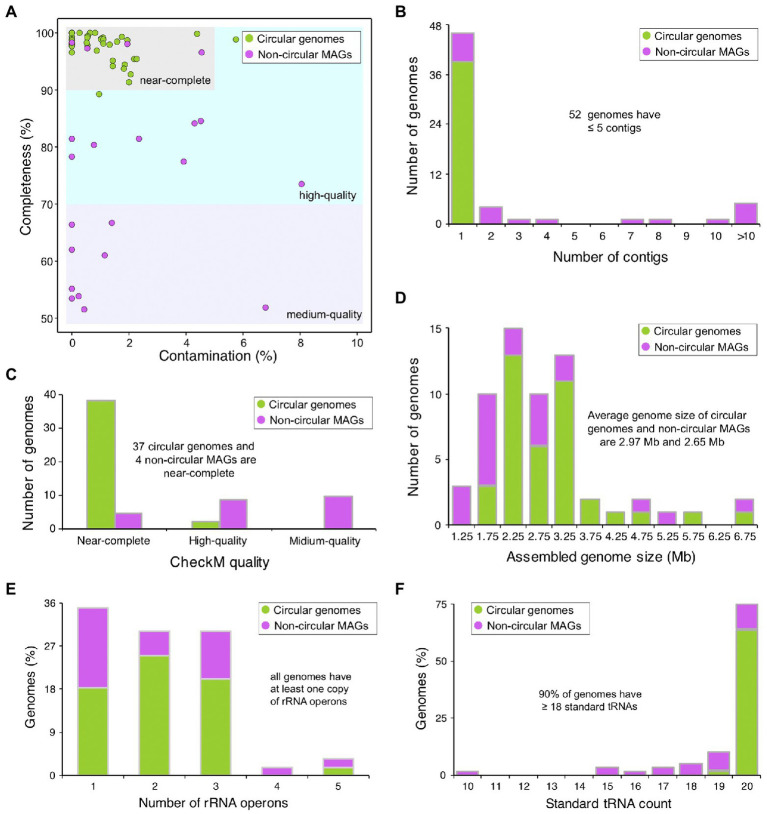
Statistics and evaluation of assembled microbial genomes. **(A)** CheckM evaluation of assembled microbial genomes. Circular genomes refer to circular contigs, and non-circular MAGs refer to genome assemblies derived from binning algorithms. Genomes are defined as “near-complete,” if their CheckM completeness values are ≥90% and contamination values are <5%, “high-quality” if completeness ≥70% and contamination <10%, or “medium-quality” if completeness ≥50% and contamination <10%. **(B–D)** Distribution of contig numbers, CheckM quality values, and genome sizes of assembled microbial genomes. **(E,F)** Distribution of the number of rRNA operons and standard tRNA genes for assembled genomes.

To construct the reference genomes of microorganisms in AD of food waste, we combined the assembled circular contigs and non-circular MAGs derived from binning algorithm. After removing the redundant and low-quality genomes, a total of 60 non-redundant microbial genomes were generated, including 39 circular microbial genomes and 21 non-circular MAGs (Figure 2A and Supplementary Table S2). Of these 60 genomes, 46 genomes contain only one contigs, six genomes contain 2–5 contigs, and only five genomes contain >10 contigs (Figure 2B), which are much superior to the MAGs derived from Illumina short reads (Campanaro et al., 2020; Ma et al., 2021). Based on the quality assessment by CheckM (Parks et al., 2015), 95% of the circular genomes are near-complete, except one genome with Cp < 90% and one genome with Ct > 5% (Figure 2C). However, only 19% of the non-circular MAGs are near-complete (Figure 2C), mostly due to the relatively low completeness of these genomes (Figure 2A), and the average genome size of non-circular MAGs (2.65 Mb) is smaller than that of circular genomes (2.97 Mb; Figure 2D).

In bacteria and archaea, the ribosomal RNA (rRNA) operon typically encodes the 16S, 23S, and 5S rRNA genes, and the rRNA operon usually exists in multiple copies, ranging from 1 to 15 in bacteria and 1 to 4 in archaea (Klappenbach et al., 2001; Stoddard et al., 2015). Transfer RNA (tRNA) genes are randomly distributed in the genome, often with redundant copies. With the advances in sequencing technologies, the presence and completeness of rRNA and tRNA genes are suggested to be an additional metric for assembly quality (Bowers et al., 2017). We predicted the rRNA and tRNA genes in the 60 non-redundant genomes, and found that all genomes have at least one copy of rRNA operon and 54 genomes have ≥18 types of standard tRNA genes (Figures 2E,F; Supplementary Table S2), suggesting that most of our assembled genomes are of high quality. Most genomes (95%) have 1–3 rRNA operons (Figure 2E), and the average rRNA operon copy number of circular genomes (2.1) is comparable to that of non-circular MAGs (2.0). In addition, more types of standard tRNA genes were identified from circular genomes (average number is 20.0) than that from non-circular MAGs (average number is 18.0), and of the 39 circular genomes, 38 have all 20 types of tRNA genes and only one genome has 19 types of tRNA genes (Figure 2F; Supplementary Table S2).

**Figure 3:**
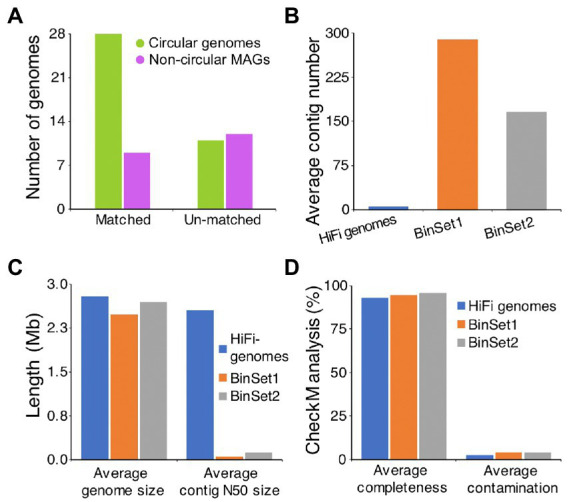
Comparison of HiFi assembled microbial genomes to the short-read assembled genomes. **(A)** Statistics of our assembled microbial genomes with matching relationship to the published short-read derived MAGs from anaerobic digestion. If one of our HiFi assembled microbial genome shows ANI value ≥95% to any of the published MAGs, it is supposed to match to the published MAG. Comparison of average contig number **(B)**, assembled genome size and contig N50 size **(C)**, and CheckM quality **(D)** between our HiFi assembled microbial genomes and the corresponded matching MAGs from two published datasets. BinSet1 published by Campanaro et al. (2020) contains 1,401 MAGs, and BinSet2 published by Ma et al. (2021) contains 2,426 MAGs.

Until now, many studies have constructed MAGs of AD from short-read assembled contigs (Treu et al., 2016; Campanaro et al., 2020). To assess the novelty and superiority of our metagenome assembled genomes, two large-scale metagenomic studies on AD were chosen for comparison. Campanaro et al. (2020) incorporated nearly 0.9 Tb of Illumina sequence data from 134 samples that published between 2014 and 2019, which representing a wide range of different biogas reactor systems, and constructed 1,401 MAGs (BinSet1). Ma et al. (2021) used 1.8 Tb of Illumina sequence data that generated from 56 full-scale biogas plants fed with diverse feedstocks, and constructed 2,426 MAGs (BinSet2). The results showed that 37 of our genomes have matched (ANI ≥ 95%) MAGs from the BinSet1 or BinSet2, and the remaining 23 genomes were not found in these two researches (ANI < 95%), including 11 circular genomes and 12 non-circular MAGs (Figure 3A). Further analysis of the published MAGs with matching relationships to our 37 genomes showed that, the average contig number of our genomes (5) is much less than that of BinSet1 (289) and BinSet2 (166), while the average contig N50 size of our genomes (2,545 Kb) is much higher than that of BinSet1 (49 Kb) and BinSet2 (123 Kb; Figures 3B,C). Besides, our 37 genomes have slightly larger average genome sizes and lower average CheckM contamination values than the matching MAGs (Figures 3C,D). However, the average CheckM completeness of our 37 genomes (92.8%) was slightly lower than that of the matching MAGs in BinSet1 (94.5%) and BinSet2 (95.6%; Figure 3D), which may be resulted from the different completeness cutoffs of 50, 70, and 80% used for our genomes, BinSet1, and BinSet2, respectively. The above results indicate obvious superiority of our HiFi assembled microbial genomes over the short-read assembled MAGs.

### The phylogeny of the genomes revealing 23 novel genome representations

**Figure 4:**
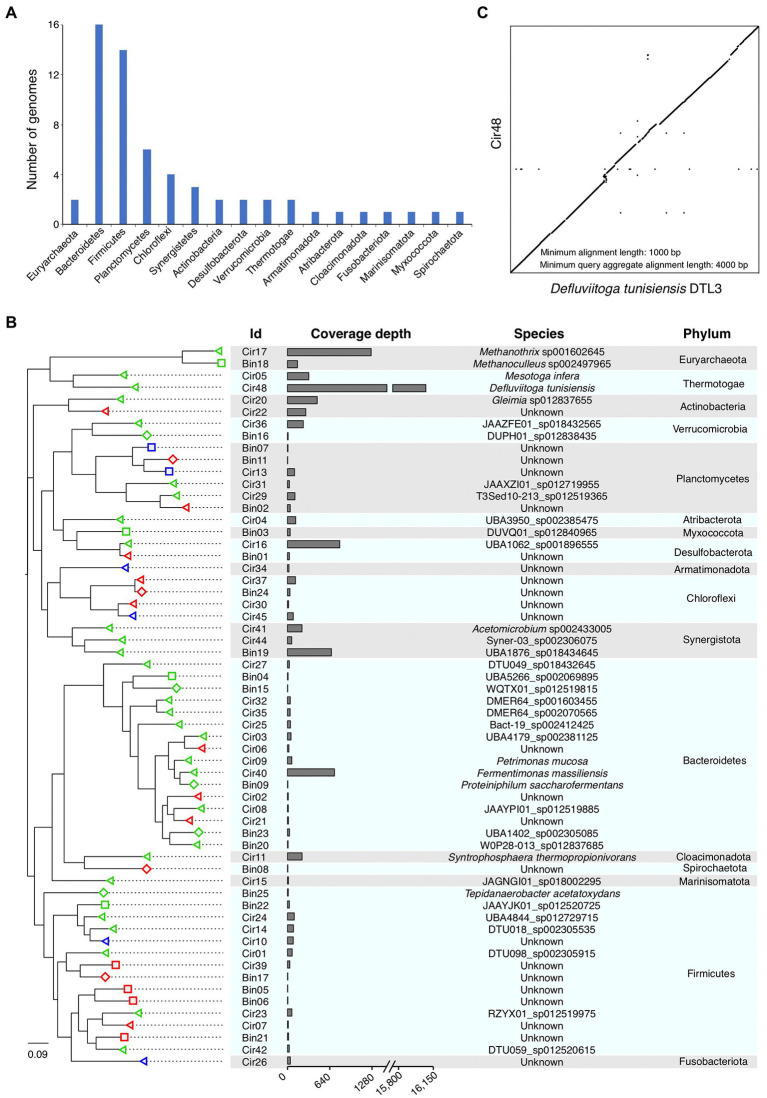
Phylogeny of the assembled microbial genomes. **(A)** Number of genomes belonged to different phyla**. (B)** Phylogenetic relationship of the assembled genomes. The phylogenetic tree was constructed using the 16S rRNA genes retrieved from all genomes, and the taxonomic annotation of the leaf nodes was performed using GTDB-Tk. The shapes of the leaf nodes correspond to the CheckM quality of the genomes, triangle represents “near-complete,” rectangle represents “high-quality,” and diamond represents “medium-quality.” The colors of the leaf nodes correspond to the taxonomic classification results of GTDB-Tk, “green” means the leaf node classified to be an existing species in the GTDB database, “red” and “blue” means the leaf node unclassified at species and genus level, respectively. Genome ids with prefix “Cir” represent circular genomes, and prefix “Bin” represents non-circular MAGs. **(C)** Pair-wise alignment of genome sequences between our genome Cir48 and the published *Defluviitoga tunisiensis* DTL3. The dot plot shows alignments longer than 1,000 bp.

We performed the taxonomic annotation of the 60 HiFi assembled genomes by using GTDB-Tk based on the GTDB database, which contains 317,542 genomes belonging to 65,703 species (Chaumeil et al., 2022; Parks et al., 2022). The results showed that only two genomes were classified as archaea (belonging to the phylum *Euryarchaeota*), while the other 58 genomes were all classified as bacteria. At phylum level, half of the 60 genomes were assigned to *Bacteroidetes* (16) and *Firmicutes* (14), followed by *Planctomycetes* (6), *Chloroflexi* (4), and *Synergistota* (3; Figure 4A). The remaining 17 genomes were classified into 12 phyla, including *Actinobacteria* (2), *Desulfobacterota* (2), *Euryarchaeota* (2), *Thermotogae* (2), *Verrucomicrobia* (2), *Armatimonadota* (1), *Atribacterota* (1), *Cloacimonadota* (1), *Fusobacteriota* (1), *Marinisomatota* (1), *Myxococcota* (1), and *Spirochaetota* (1; Figure 4A). In summary, the 60 HiFi assembled genomes come from a total of 17 phyla, revealing a high diversity of microbial genomes constructed by HiFi sequencing data.

According to the taxonomic annotation by GTDB-Tk, all genomes have been successfully classified at high taxonomic level (domain, phylum, class, order, and family), while 6 and 17 genomes cannot be classified at genus and species level, respectively (Figure 4B; Supplementary Tables S3, S4), suggesting that the 6 and 17 genomes are novel assembled genomes of these genera and species. To further classify these genomes at lower taxonomic levels, the 16S rRNA genes that identified from them were taxonomic annotated using the RDP Classifier and BLAST against the 16S rRNA database. Among the six unclassified genera, three were successfully classified at genus level by RDP Classifier, and two (Cir26 and Cir 34) of the three remaining unclassified genera showed 16S rRNA gene identities <95% (Supplementary Table S3), which is the threshold for demarcating procaryotic genus (Ludwig et al., 1998). Among 17 unclassified species, four (Cir02, Cir30, Bin02, and Bin17) showed 16S rRNA gene identities <97% (Supplementary Table S4), which is the threshold for demarcating procaryotic species (Stackebrandt and Goebel, 1994). Therefore, two and four of the HiFi assembled genomes can be treated as novel genera and species, respectively.

The genome Cir26 showed the highest 16S rRNA gene identity (88.6%) to the genus *Sebaldella*, an obligately anaerobic bacteria that could hydrolyze the glucose to produce acetate and lactic acids, and one uncultured clone of this genus was identified in a mesophilic anaerobic digester (Riviere et al., 2009; Harmon-Smith et al., 2010). The genome Cir02 and Bin17 have the highest 16S rRNA gene identity to *Paludibacter jiangxiensis* (87.5%) and *Desulfonispora thiosulfatigenes* (93.1%), and both of them were anaerobic bacteria and could produce acetate from glucose and organosulfonate taurine, respectively (Denger et al., 1999; Qiu et al., 2014). In addition, the genome Cir34, Cir30, and Bin02 have the highest 16S rRNA gene identities to the phylum WS1 (90.3%), class *Anaerolineeae* (95.8%), and class *Phycisphaerae* (92.4%), respectively, but the taxonomic annotation at lower levels of these species are unknown. The above results indicated that Cir26, Cir02, and Bin17 may provide substrates to methanogens for methane production in AD, while the functions of Cir34, Cir30, and Bin02 need further investigation.

### The most abundant microbial genome belongs to *Defluviitoga tunisiensis*

Considering the coverage depth of each genome generated by mapping the HiFi reads to these genomes using Minimap2 (Li, 2018), the most abundant genome was a circular genome Cir48 (taxonomically annotated as *Defluviitoga tunisiensis*) with the highest coverage depth of 16,076 (X), much higher than that of other genomes (< 1,270 X; Figure 4B). In 2015, the complete genome sequence of *D*. *tunisiensis* L3 was reported, consisting of a circular contig with length of 2,053,097 bp and GC content of 31.4% (Maus et al., 2015). The pair-wise sequence alignment between the genomes of Cir48 and *D*. *tunisiensis* L3 showed a clear 1-to-1 syntenic relationship between these two genomes (Figure 4C). In addition, the two genomes have similar genome size (2.18 vs. 2.05 Mb) and GC content (31.2 vs. 31.4%), and high ANI value (97.8%) and 16S rRNA gene identity (100%), suggesting that these two genomes belong to the same species of *D*. *tunisiensis*. *Defluviitoga tunisiensis* was firstly isolated from an anaerobic digester, and it can utilize a variety of complex polysaccharides, such as cellulose, chitin and xylan, and acetate, carbon dioxide and hydrogen were found as possible end-products of the fermentation process (Ben Hania et al., 2012; Maus et al., 2016). Since these compounds are essential substrates for producing of methane, it is reasonable to explain the highly abundant of Cir48 in current anaerobic digester for food waste.

The other abundant genomes include one archaeal genome Cir17 (*Methanothrix* sp., 1,268 X) and four bacterial genomes Cir16 (UBA1062 sp., 792 X), Cir40 (*Fermentinonas massiliensis*, 712 X), MAG Bin19 (UBA1976 sp., 666 X), and Cir20 (*Gleimia* sp., 451 X; Figure 4B). The genome Cir17 belongs to the archaeal phylum *Euryarchaeota*, and the coverage depth of this genome is obviously higher than that of the other archaeal genomes (Figure 4B), suggesting that *Methanothrix* sp. may play the most important role in the last step of methane production in the AD microbial community here analyzed. Noticeably, several complete circular genomes with relatively low coverage depth (<20 X) were also successfully assembled by Hifiasm-meta, including Cir02 (11 X), Cir08 (12 X), Cir07 (12 X), Cir15 (14 X), Cir21 (14 X), and Cir30 (16 X; Figure 4B).

**Figure 5:**
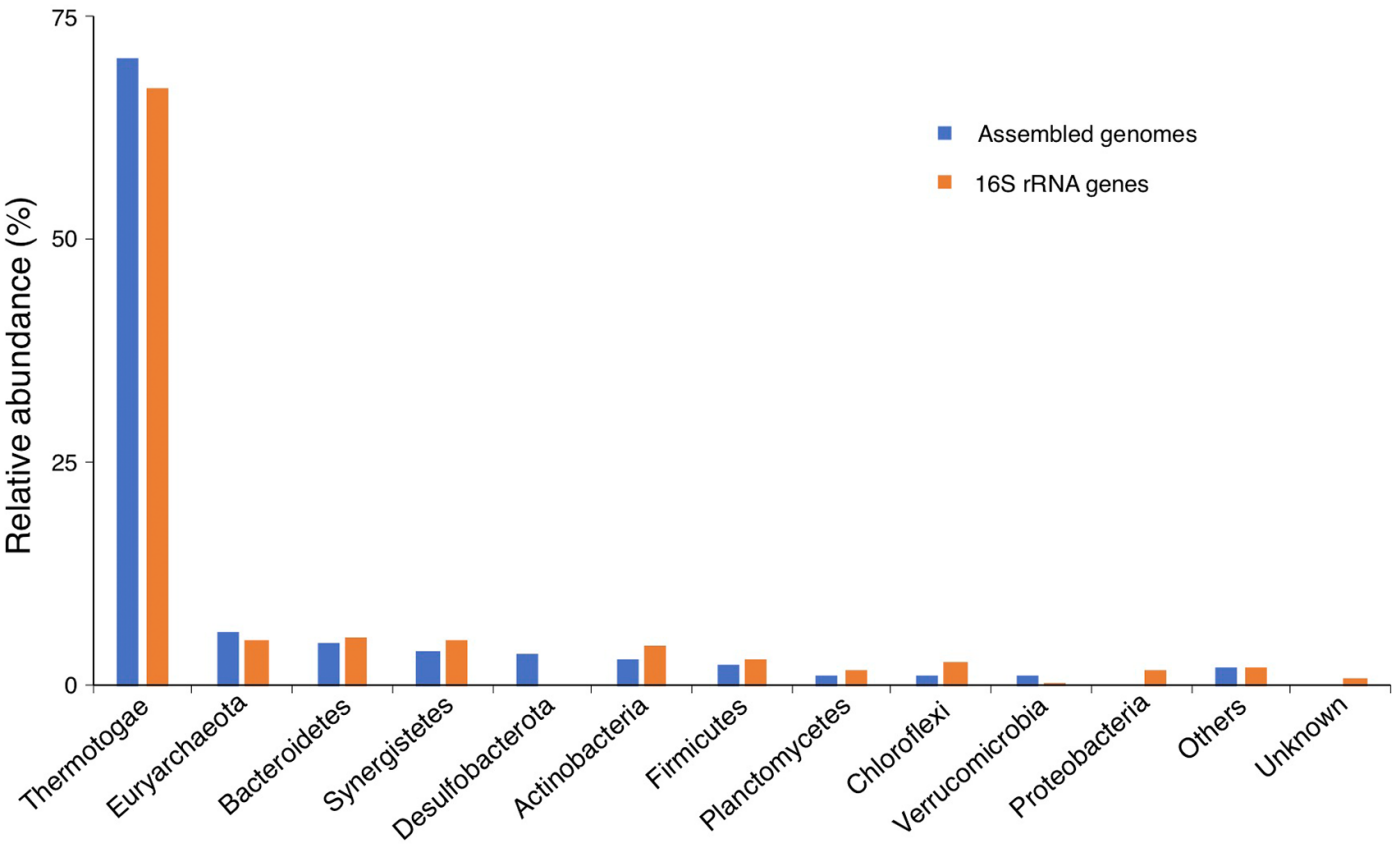
Microbial community of the anaerobic digester for food waste. The microbial community derived from the assembled genomes was generated based on the taxonomic annotation of the whole genomes by using GTDB-Tk, while that derived from the 16S rRNA genes was obtained from the taxonomic annotation of the full-length 16S rRNA genes identified from the HiFi reads, using the tool RDP Classifier.

Then, the relative abundance of different taxonomic ranks was calculated based on the taxonomic annotation and coverage depth of each genome, which showed that the microbial community consisted 93.9% of *Bacteria* and 6.1% of *Archaea* at domain level. The most predominant phylum was *Thermotogae* (70.5%), followed by *Euryarchaeota* (6.1%), *Bacteroidetes* (4.7%), *Synergistetes* (4.1%), *Desulfobacterota* (3.5%), *Actinobacteria* (3.1%), and *Firmicutes* (2.4%; Figure 5). Considering the diverse lifestyle of the members of the phylum *Thermotogae* in both thermophilic (54°C) and mesophilic (37°C) anaerobic digesters (Li et al., 2013; Campanaro et al., 2016; Stolze et al., 2016; Maus et al., 2017), the dominance of *Thermotogae* in the microbial community of AD for food waste at mesophilic condition is possibly due to environmental selection.

### Full-length 16S rRNA gene diversity analysis

**Figure 6:**
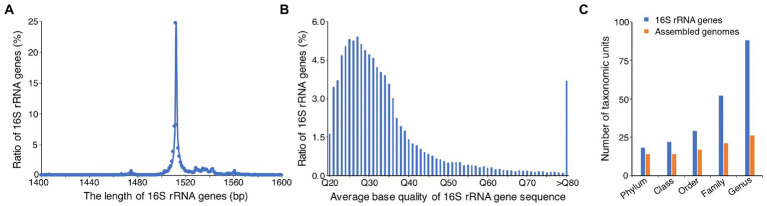
Analysis of microbial communities based on the full-length 16S rRNA genes identified from HiFi reads. **(A)** Distribution of the length of 16S rRNA genes. **(B)** Distribution of sequencing quality of 16S rRNA genes. Q20 means an average base accuracy of 99%, Q30 means 99.9%, and so on. **(C)** Comparison of the number of taxonomic units identified by genome taxonomy and full-length 16S rRNA genes at different taxonomic ranks.

To determine whether the real microbial composition of AD can be recovered from the 60 HiFi assembled genomes, we also used the full-length 16S rRNA genes identified from the HiFi reads to investigate the microbial compositions. We identified the 16S rRNA genes from the accurate HiFi long reads by INFERNAL (Nawrocki and Eddy, 2013), and found a total number of 54,135 16S rRNA genes. Unlike the previous study using the PCR amplification and PacBio HiFi sequencing to acquire the full-length 16S rRNA gene sequences (Liang et al., 2022), the present study directly identified the full-length 16S rRNA gene sequences from the PacBio HiFi long reads, without any PCR amplification. Of the identified 16S rRNA genes, most of them (67%) ranged from 1,500 to 1,520 bp, with the length peaked at 1,512 bp (Figure 6A), consistent with the reported length of microbial full-length 16S rRNA genes. In addition, the average base quality of most of the identified 16S rRNA genes (80%) ranged from Q20 (99% average base accuracy) to Q40 (99.99% average base accuracy; Figure 6B), indicating the high quality of the identified 16S rRNA genes.

Taxonomic annotation of the full-length 16S rRNA genes showed that the relative abundances of *Bacteria* and *Archaea* were 94.8 and 5.2%, respectively. The most predominant bacterial and archaeal phyla were *Thermotogae* (67.0%) and *Euryarchaeota* (5.2%), respectively, which were comparable to that derived from the assembled genomes (Figure 5). The other abundant phyla were *Bacteroidetes* (5.5%), *Synergistetes* (5.1%), *Actinobacteria* (4.6%), *Firmicutes* (3.1%), *Chloroflexi* (2.9%), *Proteobacteria* (1.8%), and *Planctomycetes* (1.7%), and they were nearly consistent with that derived from the assembled genomes, except slight differences in the relative abundance of a few phyla, such as *Actinobacteria*, *Chloroflexi*, and *Verrucomicrobia* (Figure 5). In addition, we found that the phylum *Desulfobacterota* was just identified from assembled genomes, and the phylum *Proteobacteria* was only detected from 16S rRNA genes (Figure 5), which may result from the different taxonomic annotation tools (GTDB-Tk vs. RDP Classifier) were used. We used GTDB-Tk to annotate the assembled genomes, and it classified the genomes of Bin01 and Cir16 as *Desulfobacterota*. However, when used RDP Classifier to annotate the 16S rRNA genes that identified from the two genomes, it classified the two genomes as *Proteobacteria*. So, it is hard to classify the two genomes on the basis of the current classification tools and databases. Noticeably, obviously more taxonomic units were identified by the full-length 16S rRNA genes, with the number of phyla, classes, orders, families, and genera 1.3, 1.6, 1.7, 2.5, and 3.4 times that identified by the assembled genomes, respectively (Figure 6C), revealing the advantage of using the full-length 16S rRNA gene for investigating microbial community.

## Conclusion

Given the importance of AD in treatment of food waste, we used the highly accurate HiFi long reads to investigate the microbiome in a full-scale anaerobic digester for food waste. In this study, we constructed a total of 60 non-redundant microbial genomes, including 39 circular genomes. All genomes have at least one copy of rRNA operons and 54 genomes have ≥18 types of standard tRNA genes. Noticeably, our HiFi-derived genomes exhibit substantial advantages over the previous short-read derived MAGs in continuity, completeness, and contamination metrics. Taxonomic annotation reveals that half of the 60 genomes belong to the phylum *Bacteroidetes* (16) and *Firmicutes* (14), and two and four genomes may belong to novel genus and species, respectively, which may provide substrates to methanogens for producing of methane in AD. The HiFi assembled genomes not only improves the description of the full genomes of the microbiome in AD, but also enables the discovery of novel microbial members living in this extreme environment.

Microbial community analysis based on the assembled genomes reveals the most predominant bacterial and archaeal phyla are *Thermotogae* and *Euryarchaeota*, and the genera are *Defluviitoga* and *Methanothrix*, respectively. Since 16S rRNA-based analysis is an established standard method for elucidating the composition of microbial communities, we identified 54,135 full-length 16S rRNA genes from the HiFi long reads, and revealed a consistent microbial composition with that derived from the assembled genomes, though more taxonomic units were identified. This study provides hundreds of thousands of full-length 16S rRNA genes for microbiome in AD, and deepens the understanding of the microbial community in anaerobic digester for food waste.

## Data availability statement

The datasets presented in this study can be found in online repositories. The names of the repository/repositories and accession number(s) can be found at: NCBI - PRJNA879921.

## Author contributions

FJ, QL, and TS collected the samples and performed experiments. FJ analyzed the data. HW, AW, DX, LY, LL, RC, and BY provide helpful suggestions. FJ and QL wrote the raw manuscript. YD and WF conceived the study and designed the experiments. SW, YD, and WF revised the manuscript. All authors contributed to the article and approved the submitted version.

## Funding

This project was supported by grants from Shenzhen Science and Technology Program (JCYJ20190814163805604).

## Conflict of interest

The authors declare that the research was conducted in the absence of any commercial or financial relationships that could be construed as a potential conflict of interest.

## Publisher’s note

All claims expressed in this article are solely those of the authors and do not necessarily represent those of their affiliated organizations, or those of the publisher, the editors and the reviewers. Any product that may be evaluated in this article, or claim that may be made by its manufacturer, is not guaranteed or endorsed by the publisher.

## Footnotes

^1^ https://github.com/ablab/viralVerify

## Abbreviations

AD: Anaerobic digestion
ANI: Average nucleotide identity
CCS: Circular consensus sequence
COD: Chemical oxygen demand
Cp: Completeness
Ct: Contamination
Gb: Gigabyte
HiFi: High-fidelity
Kb: Kilobase
kg: Kilogram
L: Liter
mg: Microgram
mL: Microliter
MAG: Metagenome-assembled genome
Mb: Megabase
rRNA: Ribosomal RNA
RDP: Ribosomal database project
tRNA: Transfer RNA
TAN: Total ammonia nitrogen
TS: Total solid
VFA: Volatile fatty acid
v: Volume
w: Weight
PacBio: Pacific biosciences
